# The Effect of Laparoscopic Sleeve Gastrectomy on Glycemic Control in Type 2 Diabetic Patients

**DOI:** 10.7759/cureus.16986

**Published:** 2021-08-07

**Authors:** Ali Al Khayat, Sarah Al Hendi, Iman Qadhi, Ahmad Al Murad

**Affiliations:** 1 General Surgery, Mubarak Al-Kabeer University Hospital, Kuwait City, KWT; 2 Family Medicine, Al-Surrah Clinic, Al-Asimah Health Center, Kuwait City, KWT; 3 General Surgery, Al Amiri Hospital, Kuwait City, KWT

**Keywords:** diabetes remission, type 2 diabetes, sleeve gastrectomy, bariatric surgery, obesity

## Abstract

Introduction

The prevalence of diabetes mellitus type II (T2DM) in Kuwait in 2013 was 23.09%, ranking ninth globally and second in the Middle East and North Africa (MENA) region. It’s been frequently reported as a growing public health concern. Our retrospective study will focus on the effect of laparoscopic sleeve gastrectomy (LSG) on the glycemic control of T2DM.

Methods

From December 2012 to January 2014, 70 patients with T2DM underwent LSG during the study period. A retrospective patient file review was performed and a follow-up on participants was carried out in February 2014. Fasting plasma glucose (FPG) was taken pre- and post-operatively. Patients were followed up to monitor the change in diabetic medications in terms of quantity, type and dose.

Results

The mean reduction of FPG after surgery was 2.94+3.66 (P < 0.001) over a mean interval of eight days (range, 0-34 days). Immediate reduction in FPG was seen in 61 patients (87%), and the greatest reduction was seen in the age group <40 years. Diabetes remission was seen in 49 patients (70%), while 20 (29%) had reduction in medication. All patients underwent a safe surgical procedure. There were no conversions to open surgery and no significant complications or mortalities.

Conclusions

Our study shows that LSG procedure has an immediate positive effect on the glycemic control of T2DM, in addition to the long-term evidence of complete resolution of diabetes in most patients or improvement in glycemic control, which has further highlighted the positive outcome of LSG, diminishing morbidity, risk factors, co-morbidities and health-expenditure.

## Introduction

Type II diabetes mellitus (T2DM) is a chronic metabolic disorder that is characterized by insulin resistance and insulin deficiency, either or both of which may be present at the time of diagnosis [1-3]. It is considered to be one of the most common diseases worldwide that is associated with obesity and numerous other comorbidities. In order to reduce the extent of the disease and its associated complications, improving glycemic control remains an important treatment objective in a diabetic patient [4].

Kuwait is a small country with a current population of 4,464,521, of which 30% are Kuwaiti citizens [5]. Nonetheless, the prevalence of T2DM in 2013 was 23.09%, ranking Kuwait ninth globally and second in the Middle East and North Africa (MENA) region. The healthcare system spends a mean of 1,866 US Dollars per person on diabetes-related healthcare, ranking third in the MENA region in terms of expenditure. The mortality in adults (ages 20-79) due to diabetes was 1,122 persons in 2013 [6]. T2DM has already been reported as a growing public health concern in Kuwait [7-11].

Although medical therapies and lifestyle modification methods available for glycemic control have managed DM as a condition, neither have the ability to induce remission (discontinuation of drug therapy and normalization of glycemic blood values) [12]. Recently, attention has been drawn towards surgical interventions in order to improve glycemic control as well as providing total resolution of the disease in diabetic patients. 

Sleeve gastrectomy is one of the surgical novelties for the control of obesity and its related co-morbidities. The procedure is primarily based on the reduction of gastric size whilst preserving the continuity of the gastrointestinal tract [13]. The results of the procedure are not limited to weight loss but extend to have favorable outcomes on obesity-related comorbidities: diabetes mellitus, hypertension, dyslipidemia, obstructive sleep apnea, etc [14].

This retrospective study will focus on the effect of laparoscopic sleeve gastrectomy (LSG) on T2DM based on fasting plasma glucose (FPG) level pre- and post-surgery. Additionally, change in medication of diabetes mellitus after the surgery in terms of quantity, type, and dosage was observed.

## Materials and methods

Initially, 107 patients were selected from surgical records in Al-Amiri Hospital in Kuwait with an inclusion criteria of diabetes mellitus and undergoing laparoscopic sleeve gastrectomy, however no exclusions were made at that stage. Participants were contacted by phone to obtain consent to review their medical files for research purpose and an ethical approval was obtained from ethical committee at the Ministry of Health of Kuwait. Upon receiving patient files the following exclusion criteria was used: diabetes mellitus Type 1, patient files missing/lost, significant information missing, gastric band, or gastric bypass. 

**Figure 1:**
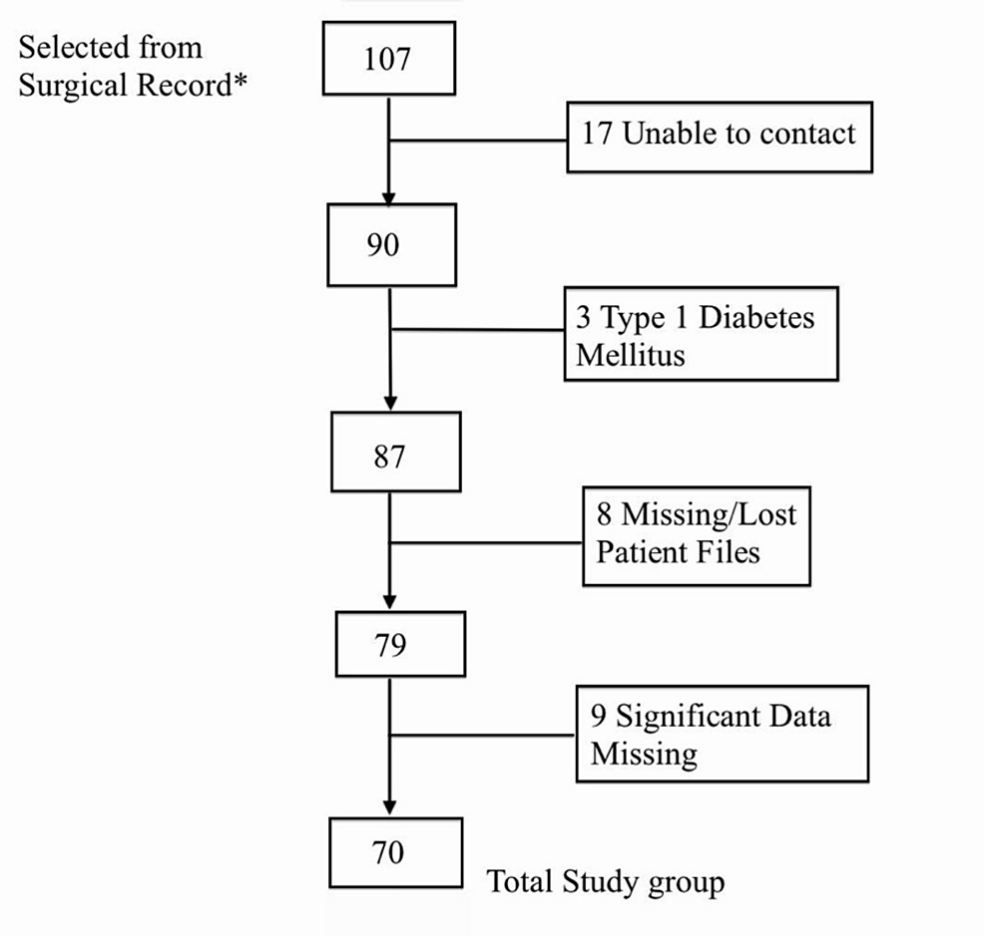
Patient Flow throughout the study

Our initial study group was reduced to 70 after being screened through the exclusion criteria. Participant’s flow throughout our research is demonstrated in Figure 1. 

Seventy patients who were affected by T2DM and its associated comorbidities underwent LSG during the study period from December 2012 to January 2014. All patients had their medical files reviewed retrospectively. In regards to glycemic control, a comparison was made between the latest FPG level within one month prior to surgery and the earliest FPG level within one month post-surgery; this approach was chosen in order to demonstrate the immediate effect of the surgical procedure on glycemic control. In addition, patients were followed up by phone mainly to determine the change of medications prescribed for T2DM, thus further demonstrating the long-term outcome of the surgery clearly.

All procedures included in the study were performed laparoscopically, in which a 36 French orogastric tube was routinely used with reinforcement of shears with a matrix of thrombin and fibrin glue. All patients underwent routine methylene blue testing intra-operatively to check for possible leakage from the staple line, and any leaking points were oversewn. A postoperative swallow test with gastrografin was not performed routinely as increasing evidence shows that it’s not required unless clinically indicated [15].

Postoperatively, a liquid diet was immediately started. Vitals were monitored, especially heart rate, as tachycardia is the first sign suggestive of complication. Patients were discharged as soon as they could ambulate, tolerate oral fluids and were cleared of significant clinical complications. Patients were advised to remain on a liquid diet for two weeks and were followed up in our outpatient clinic.

## Results

The study group consisted of 45 women (64%) and 25 men (36%) with a mean age of 43.93 years (range, 15 to 61 years), with a mean FPG preoperatively of 11.37 mmol/L (range, 4.2 to 25.2 mmol/L) and a mean BMI of 45.75 kg/m2 (range, 32.04 to 75.33 kg/m2).

Among the study participants, 51 (73%) had a BMI of > 40 kg/m2, while 19 (27%) had a BMI of between 30 and 40 kg/m2. Patients in the study group suffered from a wide range of comorbidities: hypertension, obstructive sleep apnea (OSA), hyperlipidemia, bronchial asthma, depression, ischemic heart disease, renal impairment or failure, osteoarthritis, Parkinsonism, hypothyroidism and stroke.

All patients in our study underwent a safe surgical procedure without necessity for conversion to open surgery. No complications were observed postoperatively nor were there any mortalities. The demographics of the studied group can be seen in Table 1.

**Table 1:** Personal Characteristics of Study Group

Personal Characteristics								All (N=70)													
							N			Col%											
Age																					
Min – Max							15 - 61														
Mean ± SD	43.93 ± 11.481																				
Median (IQR)							47(15)														
Age Group (Years)																					
< 40							20			(28.6)											
40 – 49							26			(37.1)											
≥ 50							24			(34.3)											
Weight (Kg)																					
Min – Max							81 - 190														
Mean ± SD			124.30 ± 25.83																		
Median (IQR)					124(36.5)																
Height (cm)																					
Min – Max						146 – 188															
Mean ± SD		164.87 ± 9.93																			
Median (IQR)				163(12.5)																	
Gender																					
Female							45			(64.3)											
Male							25			(35.7)											
Body Mass Index (BMI)																					
Min – Max		32.04 – 75.33																			
Mean ± SD	45.75 ± 8.56																				
Median (IQR)							43.60(12.1)														
Morbid obesity Class																					
Class 2 (35< BMI >40)							19			(27.1)											
Class 3 (BMI ≥ 40)							51			(72.9)											
Smoking																					
Current		13									(18.6)										
Non-Smoker	46									(65.7)											
Ex-Smoker							7			(10.0)											
Missing							4			(5.7)											

**Figure 2:**
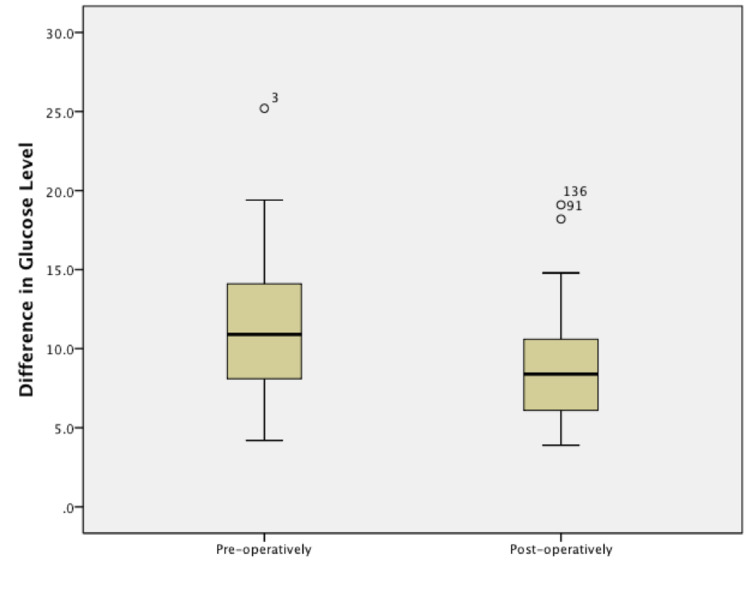
Box Plot of mean glucose levels (FPG) (mmol/L )comparing pre and post-operatively

All 70 participants experienced a safe postoperative period over a mean of 8.3 months. Follow-up mean FPG pre-operatively was 11.37+3.97 mmol/L and post-operatively 8.79+2.98 mmol/L. The mean reduction of FPG after surgery was 2.94+3.66 mmol/L (P < 0.001) over a mean interval of eight days (range, 0-34 ) (Figure 2).

**Figure 3:**
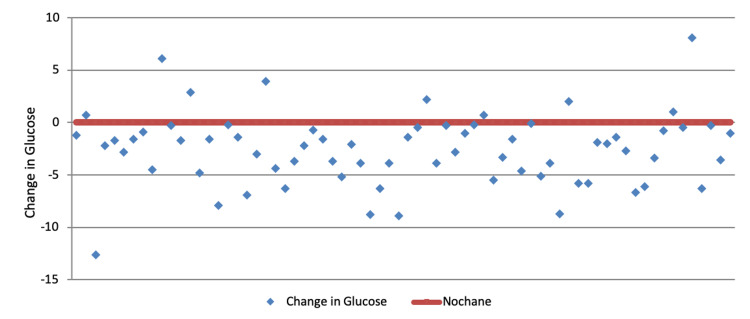
Difference of Glucose post-operatively for each participant

Furthermore, 61 (87%) showed immediate improvement of glycemic control postoperatively over a mean interval of eight days (range, 0-34). Although nine (13%) showed slight increase following surgery, however their glycemic control appears to be continuously improving thereon afterwards during follow-up (Figure 3).

**Figure 4:**
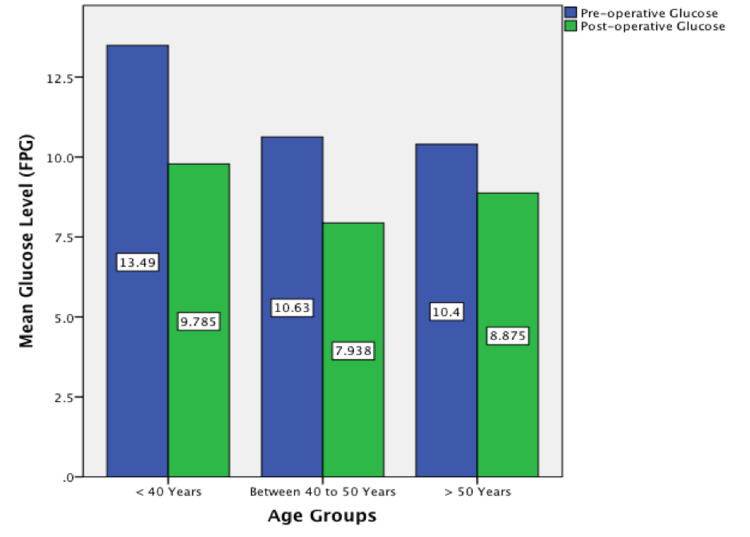
Bar chart comparing Pre and Post-operative glucose according to age groups

Figure 4 compares pre- and post-operative FPG levels by categorizing participants according to three age groups: <40, 40-49 and >50 years of age. On reviewing this table, the greatest mean reduction in FPG postoperatively was observed in the age group of <40 years.

Remarkably, 49 patients (70%) were currently free from diabetic medical therapy due to normalization of glycemic control with improvement in other co-morbidities and concurrent significant weight loss (Table 2).

**Table 2:** Frequency of Post-operative Remission of Diabetes

		All (N=70)	
	N	Col%	
Remission of Diabetes			
No	21	(30.0)	
Yes	49	(70.0)	

**Figure 5:**
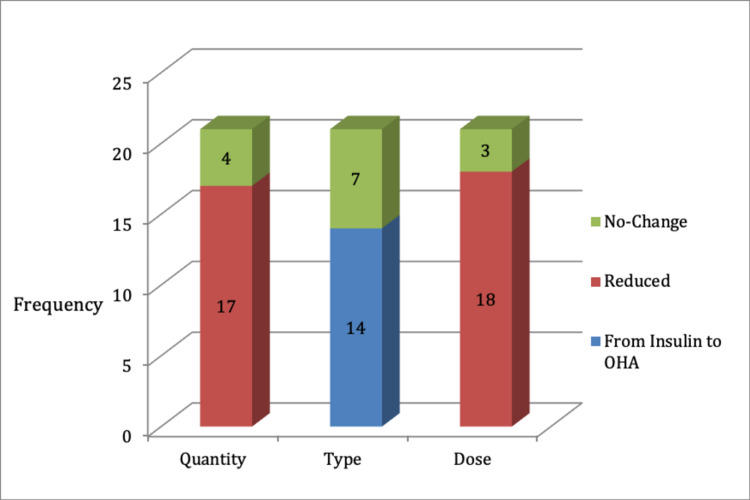
Bar chart showing frequency of different changes of medication

Nevertheless, the remaining 21 patients (30%) have still improved in glycemic control. This was more evident from the observational follow-up on their diabetic medication requirements that further substantiates the immediate positive effect. Changes in medication in terms of quantity, type, and dose, were monitored over a mean of eight months follow-up (range, 0-14 months). In 20 patients out of these 21, most patients experienced a positive change in all three categories, the remainder had a change in either a combination of the categories or at least in a single category. Further follow-up could have shown greater improvement as patients are continuously progressing positively. It is noteworthy that none of the patients experienced a negative change in medication, for example an increase in dose of diabetic medication. Figure 5 reveals that 17 patients had a reduction of medication in terms of quantity (decrease in the number of different diabetic treatments), 18 patients had a reduction of medication in terms of dose and 14 patients had switched their insulin medication to oral hypoglycemic agents (OHA).

Table 3 shows the association of the different demographic factors in relation to the remission of T2DM. Diabetes remission in relation to age groups shows a P value = 0.556, whereby in relation to gender shows a P value = 0.174 and in relation to class of morbid obesity shows P = 0.861; this indicates that there is no significant association. However, in regards to the association to gender, we can’t consider its significance, as the male to female ratio is not evenly distributed.

**Table 3:** Demographic Factors and Remission of Type 2 Diabetes Mellitus

Demographic Factors			All (N=70)					Diabetes Remission Yes No				P value	
		N			Col%			Col%		Col %			
Age Groups (Years)												0.556^2^	
< 40		20			(28.6)			6 (28.6)		14 (28.6)			
40 – 49		26			(37.1)			6 (28.6)		20 (40.8)			
≥ 50		24			(34.3)			9 (42.8)		15 (30.6)			
Gender												0.174^1^	
Male		25			(35.7)			5 (23.8)		20 (40.8)			
Female		45			(64.3)			16 (76.2)		29 (59.2)			
Morbid Obesity												0.861^2^	
Class 2 (35>BMI<40)		19			(27.1)			6 (28.6)		13 (26.5)			
Class 3 (BMI≥40)		51			(72.9)			15 (71.4)		36 (73.5)			
	^1 ^Based on chi-square test												
	^2 ^Based on chi-square test for trend												

## Discussion

Obesity is defined as an excess fat accumulation over a period of time; that has an inverse effect on health [16]. It is increasingly becoming a worldwide threat to health, as the significance of obesity relies greatly on the complications arising from its related co-morbidities. The transition from obesity to normal BMI range (18.5-24.9) [17] through diet, exercise and medication can be difficult to achieve, especially if the patient has a very high BMI; additionally, there is a great tendency of regaining weight.

Hormones that control hunger and appetite, ghrelin and leptin, have been studied in relation to diet. Obese people seem to have higher levels of these hormones rendering diet difficult and may even torment patients by starvation. Hormone levels may remain elevated even after losing weight, hence remaining vigilant is essential in weight maintenance. The maintenance phase may be indefinite [18-20].

Interestingly, there is growing evidence that the downward shift of BMI post-LSG, whereby the stomach is reduced by about 75% of its original size [13,21], is not solely dependent on the reduction of the stomach size alone but also affected by neuro-hormonal factors [22].

In Al-Amiri Hospital, the guidelines for LSG require the patients to be morbidly obese, in order to be a candidate for surgery; class III (BMI ≥ 40 kg/m2) and class II (35 ≤ BMI ≥ 39.9 kg/m2 in the presence of comorbidities) [23]. In current literature, LSG has shown excellent outcomes in terms of weight loss and improvement of obesity-related comorbidities. It has been already shown to treat comorbidities, particularly hypertension, dyslipidemia and obstructive sleep apnea [14].

The effect of LSG is not restricted to weight loss but extends to include the management or cure of concomitant comorbidities. Therefore, LSG may be proposed to treat diabetic patients with metabolic syndrome components [24]. It would be a clear-cut approach to tackle a cluster of co-morbidities that tend to cluster with T2DM, such as hypertension, dyslipidemia and abdominal obesity, which LSG has already been proven to treat effectively. This would be a very efficient and cost-effective management rather than multiple different conservative medical therapies that strain the healthcare system, which more often than not carry a high failure rate in these settings. Nevertheless, there is insufficient evidence for recommending a bariatric surgical procedure specifically for solely glycemic control, lipid lowering, or cardiovascular disease risk reduction, independent of BMI criteria [25,26].

Numerous studies assessing the effect of various types of bariatric surgery suggest that the normalization of glycemic control and remission of diabetes in moderately obese patients is probably independent from weight loss and correlated to the surgically induced modification of secretion of upper digestive gastrointestinal regulatory hormones [27-29].

Further studies focused primarily on SG, evaluating its effect on T2DM in moderately obese patients [12,30]. These studies have suggested that moderately obese patients have been observed to have complete remission of diabetes at rates of 88.8% (8 out 9) in comparison to 0% (0 out of 9) with conservative medical therapy [12]. Even though they’re only small studies, bariatric surgery including LSG appears more promising and effective than conservative medical therapy mainly because of cost reduction of medical therapies of comorbidities [31].

A systematic review assessed the effect of LSG among other bariatric surgeries on T2DM, as a low morbidity surgical procedure. Results show that most patients have experienced either resolution or improvement in DM markers after the surgery, concluding that LSG might play an important role as a metabolic therapy for patients with T2DM [32].

We believe that our research contributes valuable information that can assist in the appropriate use and net benefit of SG in a diabetic population in comparison to other bariatric surgeries. Our research has been based on a retrospective approach to assess the immediate effect of surgery on glycemic control combined with follow-up to assess the long-term benefit in reduction of medication requirement. 

A significant obstacle that hindered our study to include a larger study group was the loss of follow-up of many patients. In this study, we had to exclude 17 patients attributed to outdated contact information of patients that have not been updated. A further nine patients were excluded due to incomplete data from their files, probably due to the patient attending different public hospitals and/or between public and private hospitals, and another eight patients were excluded due to missing/lost files. Only a handful of our participants had HbA1c test results in their medical file, nearly all of which have only a single result and/or performed at a significant time interval from the surgery. The inclusion of HbA1c results in our study would have been a more reliable indicator of glycemic control than FPG.

## Conclusions

The results of our study seem to demonstrate that sleeve gastrectomy has an immediate effect on reducing FPG levels in diabetic patients thus improving their glycemic control. In addition, observational follow-up revealed the long-term outcome of the surgery and further substantiated the immediate influence on glycemic control. 

Further investigations in the difference in life expectancy and morbidity in patients on conservative medical therapy versus LSG are needed before LSG can be proposed to treat T2DM alone independent of BMI criteria. A larger number of patients are needed in the future as well as clinical prospective studies with well-designed end points in terms of T2DM, associated comorbidities and life expectancy.

LSG in diabetic individuals has proven to have significant advantages in terms of diminishing their morbidity and health risk factors by relieving diabetes and considerable associated co-morbidities, especially during a long-term period of observational follow-up. In addition, this results in reduction of medical therapy of diabetes and other co-morbidities and establishes a more cost-effective management, even if surgery appears to be more expensive in the short term than conservative medical therapy. Therefore, our study shows that surgery is a more promising and effective treatment in the long-term period than conservative medical therapy.
